# Tumor lysate-cloaked CuMOF-sorafenib nanoassembler: A synergistic cuproptosis-ferroptosis nanoweapon against tumors

**DOI:** 10.1016/j.mtbio.2025.102623

**Published:** 2025-12-05

**Authors:** Mingyue Zhang, Ke Zhang, Shiyao Guo, Peiran Chen, Bangliu Yang, Xueqian Wang, Xiaotong Lu, Yuhong Zhuo, Shaofeng Chen, Dongqin Yu, Lian-Hua Fu, Chao Qi, Kaiyong Cai

**Affiliations:** a Key Laboratory of Biorheological Science and Technology, Ministry of Education, College of Bioengineering, Chongqing University, Chongqing, 400044, China; b School of Biomedical Engineering, Shenzhen University Medical School, Shenzhen University, Shenzhen, 518055, China

**Keywords:** Ferroptosis, Cuproptosis, Tumor lysate, Metal organic frameworks, Drug delivery system

## Abstract

Clinical tumor management is hindered by insufficient therapeutic efficacy and systemic side effects. Ferroptosis and cuproptosis, emerging metal ion-disruption-driven regulated cell death pathways, hold significant therapeutic potential. However, their combined efficacy is constrained by the tumor microenvironment (TME), where elevated levels of glutathione (GSH) and glutathione peroxidase 4 (GPX4) effectively mitigate reactive oxygen species (ROS) and lipid peroxidation. To overcome these barriers, we developed a biomimetic Cu-MOF-sorafenib nanoassembler (CMSP) for synergistic cuproptosis-ferroptosis-based anticancer therapy. CMSP comprises sorafenib (SOF)-loaded Cu-MOFs coated with tumor cell lysate–derived proteins. It exerts robust antitumor effects through three interconnected mechanisms: i) The tumor lysate coating facilitates homotypic targeting and immune evasion, thereby enhancing tumor-specific accumulation; ii) The acidic TME triggers the release of copper ions, which amplify ROS generation *via* Fenton-like reactions, while SOF inhibits System Xc^−^, thereby depleting GSH and downregulating GPX4 expression; iii) Copper ions induce the aggregation of lipoylated proteins, initiating cuproptosis, while SOF synergistically promotes lipid peroxidation, driving ferroptosis. Both *in vitro* and *in vivo* studies demonstrate the efficient tumor accumulation, growth suppression, and metastasis inhibition of CMSP. By integrating biomimetic targeting, oxidative stress amplification, and dual-pathway synergy, CMSP presents a promising strategy to advance the development of personalized antitumor nanomedicines.

## Graphical abstract

A tumor lysate-coated nanoassembler (CMSP) was developed, encapsulating sorafenib within a Cu-MOF structure. The biomimetic coating enables homologous tumor targeting and confers immune evasion capabilities. In the acidic tumor microenvironment, CMSP releases Cu^+^ ions that catalyze ROS generation *via* Fenton-like reactions, as well as sorafenib, which inhibits System Xc^−^ to deplete GSH and suppress GPX4 activity. This dual mechanism acts synergistically to induce both cuproptosis and ferroptosis. Therefore, CMSP represents a promising strategy for advancing personalized antitumor nanomedicine development.

**Figure:**
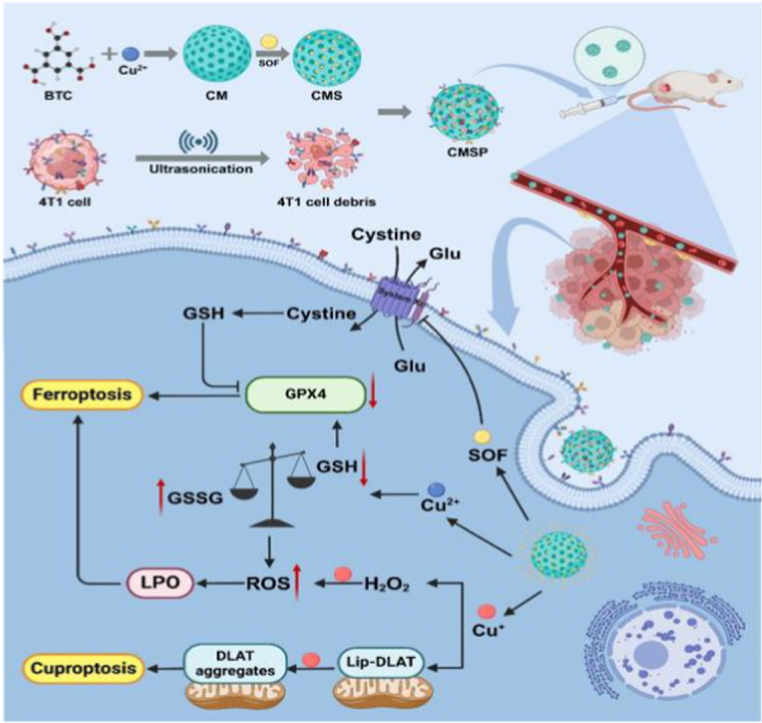

## 1 Introduction

Malignant tumors represent the second leading cause of mortality worldwide [1]. Conventional treatment modalities such as surgery, chemotherapy and radiotherapy continue to face substantial limitations, including drug resistance, off-target toxicity, and the risks of metastasis and recurrence [2]. In recent years, regulated cell death (RCD) has emerged as a major research focus owing to its controllable nature and therapeutic relevance [3,4]. Among the various subtypes of RCD, ferroptosis and cuproptosis—novel modalities mediated by the disruption of metal ion homeostasis—have shown significant promise for cancer therapy by perturbing redox homeostasis and impairing mitochondrial function, and have also been associated with immunogenic responses triggered by reactive oxygen species (ROS), such as damage-associated molecular patterns (DAMPs) release [[5], [6], [7], [8]]. Ferroptosis is characterized by iron-dependent lipid peroxidation, primarily initiated *via* Fenton-like reactions and exacerbated by inhibition of the glutathione peroxidase 4 (GPX4)-mediated antioxidant defense system [[9], [10], [11]]. Cuproptosis is triggered by excessive Cu^+^ ions bind to lipoylated proteins in the tricarboxylic acid cycle (TCA), inducing their toxic aggregation and depletion of Fe-S cluster proteins, ultimately leading to mitochondrial dysfunction [[12], [13], [14], [15], [16], [17], [18]]. Notably, sorafenib (SOF), an FDA-approved multi-kinase inhibitor used in systemic liver cancer therapy, is a well-established inducer of ferroptosis [19,20]. When co-administered with copper ion ionophores, SOF enhances protein lipoylation by inhibiting the mitochondrial matrix protease-mediated degradation of ferredoxin 1 (FDX1), while simultaneously suppressing the synthesis of glutathione (GSH), an intracellular copper chelator, by blocking cystine uptake [21]. These mechanisms highlight a significant crosstalk between cuproptosis and ferroptosis pathways, suggesting that their synergistic activation may enhance antitumor efficacy. However, the therapeutic potential of both pathways is significantly hindered by the tumor microenvironment (TME), where elevated levels of GSH and GPX4 efficiently neutralize reactive oxygen species (ROS) and lipid peroxides, thereby attenuating ferroptosis and cuproptosis [22,23]. Consequently, designing multifunctional nanomedicines capable of simultaneously generating ROS and depleting GSH and GPX4 holds considerable potential for overcoming these limitations [[24], [25], [26]].

Metal-organic frameworks (MOFs) are a class of porous crystalline materials with well-defined periodic structures, formed through coordination bonds between metal ions and organic linkers [27]. Their high porosity, tunable structural and functional properties, and responsiveness to environmental stimuli contribute to their broad applications in nanomedicine [[28], [29], [30]]. Similar multifunctional nanoplatform strategies have also been successfully applied to integrate structure-activity regulation, immune activation, and degradability for enhanced tumor suppression [31]. Copper-based MOFs (Cu-MOFs), which feature copper ion nodes, offer dual therapeutic advantages: they release copper ions under the acidic TME and promote ferroptosis *via* copper-catalyzed Fenton-like reactions, positioning them as effective anti-tumor drug delivery systems [[32], [33], [34]]. While the porous architecture of MOFs enables high drug loading capacity and nanomedicine exploits the enhanced permeability and retention (EPR) effect to improve tumor accumulation, conventional synthetic nanocarriers still encounter significant limitations [[35], [36], [37]]. These include rapid clearance, poor tumor tissue penetration, and insufficient targeting specificity, all of which contribute to suboptimal tumor accumulation [38]. Therefore, improving delivery efficiency remains a central challenge in enhancing anti-tumor therapeutic outcomes and minimizing systemic toxicity [39,40].

Recently, the emergence of tumor cell membrane biomimetic coating technology provides a highly promising and innovative strategy to overcome these limitations [[41], [42], [43], [44], [45]]. This approach employs membrane structures or lysates derived from tumor cells as natural “camouflage shells” to coat synthetic nanocarriers, forming hybrid systems that integrate artificial carrier functions with complex biological interfaces [[46], [47], [48]]. This strategy preserves the complete surface proteome of the source tumor cells, including adhesion molecules and homing receptors [49], as similarly noted in intact membrane-coated systems [50]. These biomolecules endow the nanocarriers with homologous targeting capabilities through homotypic binding interactions, enabling efficient recognition and anchoring to homologous tumor cells and metastatic sites [51]. Concurrently, “self” markers (e.g., CD47) present on the membrane significantly suppress macrophage phagocytosis, thereby extending circulation time and enhancing tumor accumulation [52,53]. Importantly, tumor cell lysates also retain cytoplasmic components such as intracellular proteins, nucleic acids, and tumor-associated antigens. These molecules not only contribute to the targeting function but also serve as endogenous immune adjuvants, enabling activation of anti-tumor immune responses [54]. Nanocarriers coated with tumor cell membranes or lysates can not only penetrate biological barriers and precisely target tumor tissues, but also integrate diagnostic, therapeutic, and immunomodulatory functions in a deeply synergistic manner [55,56]. This makes them highly versatile platforms for targeted drug and gene delivery, multimodal imaging, photothermal and photodynamic therapy, as well as the development of personalized cancer vaccines [57].

**Scheme 1:**
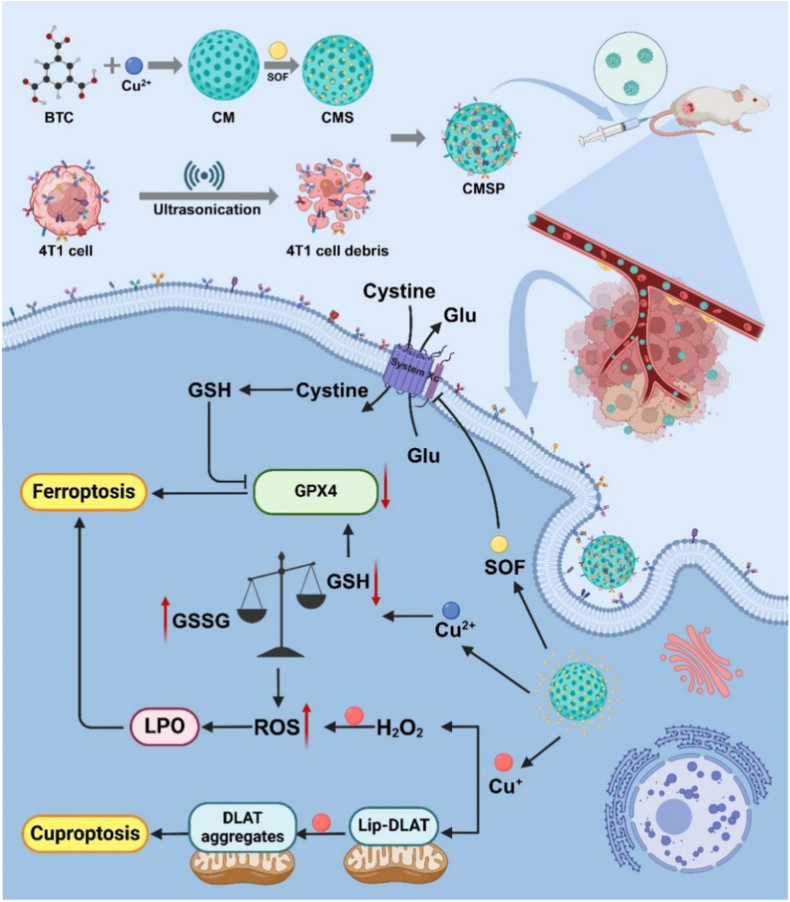
Schematic illustration of the CMSP drug delivery system for synergistic cuproptosis-ferroptosis anticancer therapy. Schemes created with BioRender.com.

Here, we constructed a tumor cell lysate-based biomimetic nano-delivery system (CMSP) by encapsulating the ferroptosis inducer SOF within a Cu-MOF, which was further coated with whole-cell lysates from 4T1 breast cancer cells (Scheme 1). This system enables synergistic induction of both ferroptosis and cuproptosis. Under the weakly acidic TME, CMSP rapidly releases SOF and Cu^2+^ ions. SOF inhibits cystine uptake, thereby reducing GSH synthesis, while Cu^2+^ is reduced to Cu^+^. The generated Cu^+^ catalyzes ROS production, inducing ferroptosis, and simultaneously binds to lipoylated proteins in the TCA cycle, disrupting Fe-S cluster protein biogenesis and triggering cuproptosis. Experimental results confirmed the successful fabrication of CMSP, demonstrating its acid-responsive behavior and GSH depletion capacity. CMSP significantly reduced the viability of 4T1 cells and induced cell death *in vitro*. And it markedly suppressed tumor growth and metastasis without significant systemic toxicity *in vivo.* These findings highlight the CMSP nanoassembler as a multi-mechanistic and tumor-targeted therapeutic system, capable of exploiting convergent oxidative stress pathways to achieve potent and selective cancer cell eradication. This work presents a promising strategy for high-efficiency, multi-pathway–integrated cancer therapy.

## 2 Experimental methods

### 2.1 Synthesis of Cu-MOF (CM)

A beaker containing 4.2 mL of initial solution (DMF:ethanol = 3:1) was heated at 50 °C under stirring. Simultaneously, 40 mL of copper(II) acetate monohydrate stock solution (DMF:ethanol = 1:1, 15 mM) and 40 mL of 1,3,5-benzenetricarboxylic acid (BTC) stock solution (DMF, 10 mM) were added dropwise over 20 min. Post-reaction, the product was collected by centrifugation, washed sequentially twice with DMF and ethanol, and dispersed in ethanol. The resulting Cu-MOF was stored at 4 °C for subsequent use.

### 2.2 Preparation of Cu-MOF/SOF (CMS)

200 mg of CM nanoparticles (NPs) were dispersed in 40 mL methanol under agitation. Subsequently, 40 mg of sorafenib powder was added and the mixture was stirred at room temperature for 12 h. The product was isolated by centrifugation, washed twice with methanol and ethanol, and redispersed in ethanol for storage at 4 °C.

### 2.3 Cell lysate extraction

4T1 cells were cultured to confluence, trypsinized, and pelleted. Cell suspensions were sonicated on ice, followed by centrifugation at 10,000 rpm for 10 min at 4 °C. The supernatant was collected, with protein concentration determined using a Bradford assay kit and adjusted to 1 mg/mL before storage at −80 °C.

### 2.4 Preparation of CMSP NPs

30.0 mg of CMS NPs were introduced into 5 mL of 4T1 whole-cell protein solution (1 mg/mL). After 15 min of room-temperature sonication, the mixture was purified *via* centrifugation (11,000 rpm, 4 °C). The resulting CMSP NPs were dispersed in saline and stored at 4 °C.

### 2.5 GSH depletion capacity

A GSH standard curve was established using a commercial kit. CM, CMS, and CMSP suspensions (0、80、160、240、320、400 mg/mL) were mixed 1:1 with GSH solution (1.1 mg/mL), incubated at 37 °C for 30 min, and centrifuged. Supernatant GSH levels were quantified using a Reduced Glutathione Assay Kit (Solarbio, BC1175).

### 2.6 TMB chromogenic assay

Suspensions of CM/CMS/CMSP (1 mg/mL), TMB (10 mM), GSH (10 mM), saline buffers (pH 7.4/6.5/5.5), and H_2_O_2_ (10 mM) were prepared. Reaction mixtures were incubated at 37 °C for 10 min at varying pH and analyzed spectrophotometrically (300–800 nm). Catalytic activity was evaluated under pH 6.5 across eight experimental groups: 1: TMB; 2: TMB + GSH; 3: TMB + GSH + CMSP; 4: TMB + GSH + H_2_O_2_; 5: TMB + H_2_O_2_ + CMSP; 6: TMB + GSH + H_2_O_2_ + CM; 7: TMB + GSH + H_2_O_2_ + CMS; 8: TMB + GSH + H_2_O_2_ + CMSP.

### 2.7 Intracellular ROS detection

4T1 cells were seeded at 1 × 10^5^ cells/well in 12-well plates and treated with control, CM (100 μg/mL), CMS (100 μg/mL), or CMSP (100 μg/mL) for 12 h. Cells were then incubated with 10 μM DCFH-DA (Beyotime, S0033) at 37 °C for 30 min in the dark, followed by three washes with PBS. ROS levels were visualized by fluorescence microscopy and quantified by flow cytometry (FITC channel).

### 2.8 Live/dead cell staining

To assess cell viability, 4T1 cells were treated with the indicated materials for 12 h. After washing with PBS, cells were incubated with Calcein-AM (2 μM) and propidium iodide (PI, 4.5 μM) from a live/dead staining kit (Beyotime, C2015M) at 37 °C for 30 min. Samples were washed gently and imaged under a fluorescence microscope (Calcein: green, PI: red).

### 2.9 Cell viability assay

4T1 or L929 cells were seeded at 5 × 10^3^ cells/well in 96-well plates and allowed to adhere overnight. Cells were treated with varying concentrations (0–140 μg/mL) of CM, CMS, or CMSP for 24 h. After treatment, 10 μL of CCK-8 reagent (Dojindo, CK04) was added to each well and incubated for 2 h. Absorbance at 450 nm was measured using a microplate reader (BioTek). Cell viability was expressed relative to the control.

### 2.10 Colony formation assay

4T1 cells were seeded into 6-well plates at a density of 500 cells/well and treated with control, CM, CMS, or CMSP (100 μg/mL) for 24 h. Cells were then cultured in fresh medium for 10 days, with medium changed every 3 days. Colonies were fixed with 4 % paraformaldehyde for 15 min and stained with 0.1 % crystal violet for 30 min. Colonies (>50 cells) were imaged and counted manually.

### 2.11 JC-1 assay for mitochondrial membrane potential

4T1 cells were seeded in 12-well plates and treated with the indicated formulations (100 μg/mL) for 12 h. Cells were incubated with JC-1 dye (5 μg/mL, Beyotime, C2006) at 37 °C for 20 min, then washed with JC-1 buffer and observed under a fluorescence microscope. Red (J-aggregates) and green (monomers) fluorescence were measured to assess mitochondrial depolarization.

### 2.12 EdU proliferation assay

4T1 cells were seeded at 1 × 10^5^ cells/well in 12-well plates and treated with CM, CMS, or CMSP (100 μg/mL) for 24 h. Cells were incubated with 50 μM EdU (Beyotime, C10310) for 2 h, fixed with 4 % paraformaldehyde, and permeabilized with 0.3 % Triton X-100.

### 2.13 Trypan blue exclusion assay

Following 24 h treatment with the materials (100 μg/mL), 4T1 cells were harvested, washed with PBS, and stained with 0.4 % trypan blue (Gibco) at a 1:1 ratio. Cells were counted using a hemocytometer. Viable (unstained) and non-viable (blue-stained) cells were quantified.

### 2.14 Cell death pathway validation

4T1 cells (10^5^ cells/mL, 200 μL/well) were plated in 48-well plates. After 24 h, cells were pretreated for 6 h with: Chloroquine (autophagy inhibitor, 25 μM), Ammonium tetrathiomolybdate (cuproptosis inhibitor, 100 μM), Ferrostatin-1 (ferroptosis inhibitor, 10 μM), Z-FA-FMK (apoptosis inhibitor, 10 μM), TCEP (disulfidptosis inhibitor, 1 mM) respectively, and CMSP (0/60/120 μg/mL) was then added for 24 h. Cell viability was assessed *via* CCK-8 assay after 30 min incubation (450 nm).

### 2.15 Cellular GSH & SLC7A11 analysis

4T1 cells (5 × 10^5^/mL for GSH; 10^5^/mL for SLC7A11) at 60–70 % confluence were treated for 12 h with: control, CM (100 μg/mL), CMS (100 μg/mL), or CMSP (100 μg/mL). For GSH measurement, cells were lysed and analyzed using a GSH/GSSG Assay Kit (Beyotime, S0053). For SLC7A11 detection, immunofluorescence staining was performed followed by flow cytometry and confocal microscopy.

### 2.16 Western blot analysis of DLAT

After treatment with materials (100 μg/mL) for 12 h, 4T1 cells were lysed in RIPA buffer supplemented with protease inhibitors. Protein concentrations were determined by BCA assay. Equal amounts of protein were resolved on SDS-PAGE gels and transferred to PVDF membranes. Membranes were blocked with 5 % BSA and incubated overnight at 4 °C with primary antibodies against DLAT (Proteintech, 16105-1-AP) and β-actin (Abcam, ab8226), followed by HRP-conjugated secondary antibodies. Bands were detected using ECL substrate and quantified by ImageJ.

### 2.17 Orthotopic tumor model

Female BALB/c mice (6 weeks old) were obtained from Chongqing Ensiweier Biotechnology Co., Ltd., with all experimental procedures approved by the Animal Ethics Committee of Chongqing University (Approval No. CQU-IACUC-RE-202504-017). A suspension of 4T1 cells in the logarithmic growth phase with high viability was prepared. Approximately 10^5^–10^6^ cells in 100 μL were subcutaneously injected into the fourth mammary fat pad (inguinal region) of anesthetized 6–8-week-old female BALB/c mice. Post-injection, mice were monitored daily, and tumor dimensions were regularly measured using vernier calipers. Tumor volume (V) was calculated as V = 0.5 × L × W^2^, where L represents the longest diameter and W the perpendicular width. Treatment commenced when tumors reached a volume of 50 mm^3^.

### 2.18 *In vivo* fluorescence imaging

To evaluate the biodistribution and tumor-targeting capability of the nanoparticles, *in vivo* fluorescence imaging was performed using ICG-labeled formulations. CMS and CMSP nanoparticles were labeled by incubating with indocyanine green (ICG, 1 mg/mL) under gentle stirring for 4 h in the dark, followed by ultrafiltration to remove unbound dye. Female BALB/c mice bearing 4T1 tumors (approximately 100 mm^3^) were intravenously injected with 200 μL of ICG-labeled CMS or CMSP (equivalent to 1 mg/kg of ICG). Fluorescence imaging was performed at 0 h, 3 h, 6 h, 9 h, 12 h post-injection using a small animal imaging system (e.g., PerkinElmer IVIS Spectrum) with excitation/emission filters set at 745/820 nm. After imaging, mice were euthanized, and major organs (heart, liver, spleen, lungs, kidneys) and tumors were excised for *ex vivo* fluorescence imaging to assess nanoparticle distribution. Signal intensity was quantified using the instrument's built-in software.

### 2.19 *In vivo* therapeutic assessment

Tumor-bearing mice (n = 5/group) received intravenous injections every other day for 21 days: saline, CM (20 mg/kg), CMS (20 mg/kg), or CMSP (20 mg/kg). Tumor volume and body weight were tracked. Post-treatment, tumors and organs were harvested for histopathology and biodistribution analysis.

### 2.20 Lung metastasis quantification

India Ink Lung Perfusion for Detecting Pulmonary Metastasis in Mice: At the end of the treatment period, mice were euthanized *via* isoflurane overdose. Following dissection to expose the thoracic cavity, a diluted India ink solution (India ink mixed with saline at a 1:10 ratio) was slowly perfused into the lungs *via* the trachea using a sterile syringe. Subsequently, the lungs were excised and rinsed with PBS to remove residual ink from the surface. The lung tissue was then fixed by immersion in Fekete's solution (a mixture of 70 % ethanol, 10 % formaldehyde, and 5 % glacial acetic acid) for 24 h. After fixation, the lungs were rinsed with distilled water, transferred to a Petri dish, and examined. The number and size of the white metastatic foci were visually assessed and counted.

### 2.21 Histopathological evaluation

Following the completion of the treatment period, mice were euthanized *via* isoflurane overdose. For histopathological analysis, tumor tissues and organs were harvested, fixed in 4 % paraformaldehyde for 24–48 h, dehydrated, paraffin-embedded, and sectioned. Consecutive sections underwent TUNEL staining, Ki67 immunohistochemistry, and immunofluorescence staining for DLAT, FDX1, SLC7A11, and GPX4, with all stained sections imaged and analyzed using fluorescence microscopy. Statistical analysis of metastatic foci number and distribution was performed to evaluate metastatic potential and treatment efficacy.

### 2.22 Hematological and serum biochemical analysis

To assess systemic toxicity, hematological and serum biochemical evaluations were conducted in healthy (non–tumor-bearing) female BALB/c mice (6–8 weeks old). Mice received intravenous injections of the corresponding nanoparticle formulations at therapeutic doses. Blood samples were collected *via* retro-orbital bleeding on days 11 and 21 post-injection. Complete blood counts (CBC) were performed using an automated hematology analyzer. Serum was isolated for biochemical analysis of liver function markers (e.g., ALT, AST, ALP, TBIL) and kidney function markers (e.g., BUN, CREA, UA) using a standard clinical chemistry analyzer.

### 2.23 Statistical analysis

Data (n ≥ 3) are expressed as mean ± SD. Analyses employed GraphPad Prism 8.0 using one-way ANOVA followed by Tukey's post hoc test for multiple group comparisons and used two-tailed unpaired Student's t-tests for comparisons between two groups. Significance levels: ∗*p* < 0.05, ∗∗*p* < 0.01, ∗∗∗*p* < 0.001, ∗∗∗∗*p* < 0.0001, ns (*p* > 0.05).

## 3 Results and discussion

### 3.1 Synthesis and characterization of CMSP

**Fig. 1:**
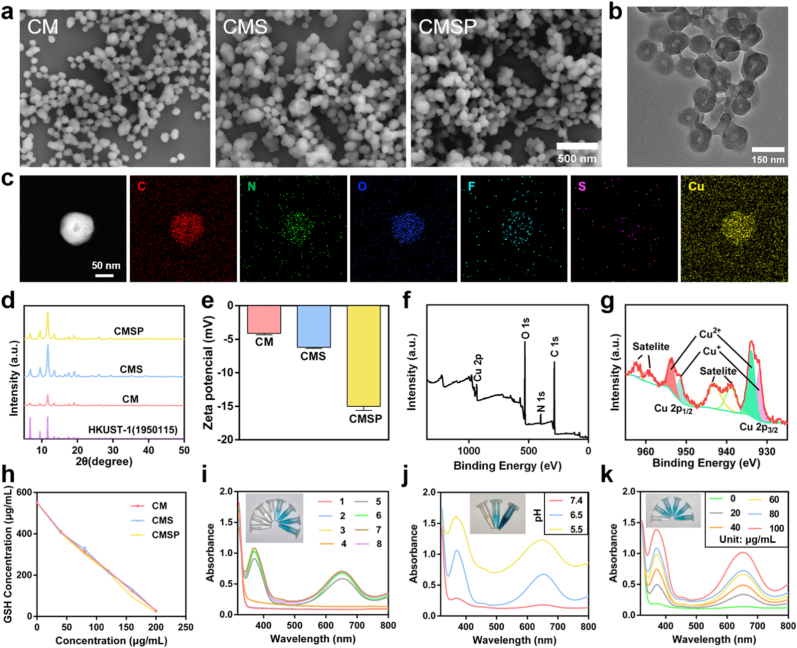
Synthesis and characterization of CMSP. (a) SEM images of CM, CMS and CMSP. TEM image (b) and elemental mapping (c) of CMSP. (d) XRD pattern of CM, CMS and CMSP. (e) Zeta potential analysis of CM, CMS and CMSP. (f) XPS full spectrum of CMSP. (g) Valence analysis of Cu on the surface of CMSP. (h) Glutathione consumption curves of CM, CMS and CMSP. (i) UV–vis absorption and photo (inset) of TMB solution after different treatments (1: TMB; 2: TMB + GSH; 3: TMB + GSH + CMSP; 4: TMB + GSH + H_2_O_2_; 5: TMB + H_2_O_2_ + CMSP; 6: TMB + GSH + H_2_O_2_ + CM; 7: TMB + GSH + H_2_O_2_ + CMS; 8: TMB + GSH + H_2_O_2_ + CMSP). UV–vis absorption and photo (inset) of TMB solution after treatment at (j) different pH values and (k) different CMSP concentrations.

Cu-MOF nanoparticles (denoted as CM NPs) were synthesized using the hydrothermal method. Subsequently, the ferroptosis-inducing agent SOF was loaded into the pores of CM, yielding Cu-MOF/SOF (CMS). Finally, total protein extracted from 4T1 cells was coated onto the surface of CMS to prepare CMSP. Scanning electron microscopy (SEM, Fig. 1a) revealed that CM exhibits a uniform spherical morphology. Neither the loading of SOF nor the subsequent coating with 4T1 total protein induced significant morphological alterations. Transmission electron microscopy (TEM, Fig. 1b) imaging showed an average particle size of approximately 100 nm. TEM elemental mapping (Fig. 1c) confirmed the presence of C, N, O, F, S, and Cu in CMSP. The F signal originates from SOF, while the S signal is attributed to the 4T1 total protein, collectively indicating the successful incorporation of both components into CMSP. Powder X-ray diffraction (PXRD, Fig. 1d) analysis demonstrated that CM, CMS, and CMSP all display characteristic peaks consistent with HKUST-1, confirming that the crystallinity of the Cu-MOF remained intact throughout the synthesis process. Dynamic light scattering (DLS, Fig. S1) measurements revealed a hydrodynamic diameter of 284.81 ± 5.01 nm for CM. The loading of SOF had minimal effect on particle size (278.32 ± 7.46 nm). However, coating with 4T1 total protein increased the hydrodynamic diameter to 308.61 ± 5.05 nm. Zeta potential measurements (Fig. 1e) showed negative surface charges for CM, CMS, and CMSP, with values of −4.11 ± 0.22 mV, −6.20 ± 0.15 mV, and −15.00 ± 0.61 mV, respectively. The slight negativity of CM is attributed to residual carboxyl or hydroxyl groups on the MOF surface. Upon sorafenib loading, the incorporation of hydrophobic and negatively charged drug molecules further reduced the zeta potential. The pronounced shift observed in CMSP is due to the presence of negatively charged biomolecules such as phospholipids and sialic acids from the tumor cell membrane coating. This stepwise increase in surface negativity confirms the successful fabrication and biomimetic functionalization of the nanoformulations, while also favoring prolonged blood circulation and enhanced tumor accumulation. To verify the encapsulation of 4T1 tumor lysate, we performed SDS-PAGE and Bradford protein assays, which confirmed the successful surface coating of CMSP nanoparticles (Fig. S2), with an encapsulation efficiency of 54.53 ± 0.68 %. X-ray photoelectron spectroscopy (XPS, Fig. 1f) confirmed the presence of Cu species in CMSP. Deconvolution of the Cu 2p spectra (Fig. 1g) revealed peaks corresponding to Cu^2+^ at 953.7 eV and 933.9 eV, and Cu^+^ at 951.5 eV and 932.0 eV. Based on peak area analysis, the relative proportions of Cu^2+^ and Cu^+^ were calculated as 53.54 % and 46.46 %, respectively. This mixed-valence state likely arises from the intrinsic redox behavior of the Cu-MOF framework during synthesis, where partial reduction of Cu^2+^ occurs under solvothermal conditions in the presence of organic linkers. Ultraviolet–visible (UV–Vis) absorption spectroscopy was employed to quantify the SOF loading capacity in CMS (Fig. S3), revealing a drug loading efficiency of 8.90 % (w/w) for the CMS nanoplatform. Collectively, these characterization results confirm the successful preparation of the materials and the effective encapsulation of the therapeutic agent.

We hypothesize that the CMSP nanoplatform can degrade and release Cu^2+^ and Cu ^+^ ions within the weakly acidic TME. The released Cu^2+^ ions are subsequently reduced to Cu ^+^ by intracellular GSH, which then catalyzes a Fenton-like reaction to convert H_2_O_2_ into highly reactive hydroxyl radicals (^•^OH), resulting in substantial accumulation of lipid hydroperoxides (LPO) and ultimately triggering ferroptosis. To assess this mechanism, we evaluated the GSH depletion capability of CM, CMS, and CMSP. As shown in Fig. 1h, GSH concentration decreased in a dose-dependent manner for all three materials, with no significant differences observed among them. This indicates that GSH depletion is efficiently achieved, predominantly through the action of Cu^2+^ ions within the MOF structure. The Fenton-like catalytic activity of the copper species was further confirmed *via* a TMB oxidation assay. As shown in Fig. 1i, only the Cu-MOF-containing groups exhibited catalytic activity, while the control groups showed no effect. Moreover, the catalytic activity was enhanced under acidic conditions. This pH-dependent behavior is attributed to the pH-responsive nature of CM; it degrades more rapidly in acidic environments, releasing copper ions that accelerate the catalytic reaction (Fig. 1j). Additionally, higher concentrations of the CMSP led to an increased rate of the catalytic reaction (Fig. 1k).

### 3.2 *In vitro* biological evaluation

Next, we evaluated the cytotoxicity and therapeutic efficacy of CM, CMS, and CMSP against 4T1 tumor cells. First, cellular uptake was assessed using fluorescence imaging. As shown in Fig. S4, the red fluorescence intensity was significantly higher in the CMSP group compared to CMS and increased progressively over time. This suggests that CMSP is more efficiently internalized by cells, likely due to the homologous recognition and homing properties conferred by the 4T1 total protein coating. Flow cytometry quantification yielded consistent results, confirming that surface modification with 4T1 total protein significantly enhances the selective uptake of CMSP by 4T1 cells.

**Fig. 2:**
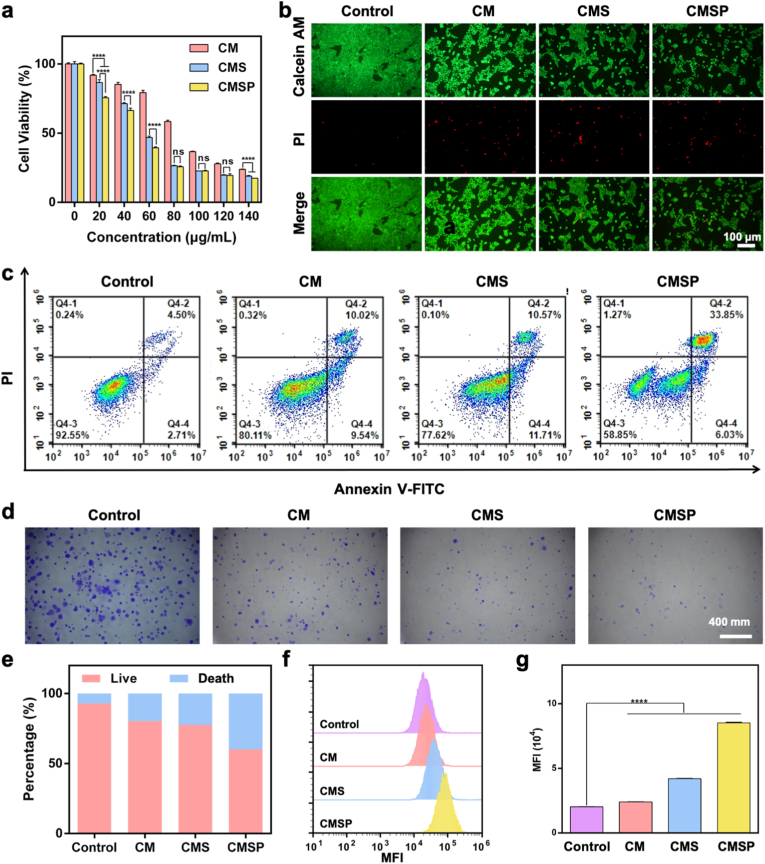
CMSP induces 4T1 cell death and suppresses proliferation. (a) Toxicity assessment of CM, CMS, and CMSP at varying concentrations against 4T1 cells. (b) Live/dead staining of 4T1 cells following 24 h incubation with 100 μg/mL CM, CMS, and CMSP NPs. (c) Flow cytometry analysis of different death stages in 4T1 cells after 12 h incubation with 100 μg/mL CM, CMS, and CMSP NPs. (d) Representative images of 4T1 cell colony formation after 12 h incubation with CM, CMS, and CMSP. (e) Quantitative statistical analysis of the flow cytometry results shown in panel (c). (f) Flow cytometry analysis and (g) corresponding quantitative statistics of 4T1 cell proliferation using CFDA SE staining after 12 h incubation with CM, CMS, and CMSP. Data represent mean ± SD, n = 3, ns: not significant, ∗∗∗∗*p* < 0.0001.

Subsequently, CCK-8 assays demonstrated that CM, CMS, and CMSP effectively inhibited cell viability and proliferation in a concentration-dependent manner (Fig. 2a). Within the 20–60 μg/mL concentration range, CMSP exhibited superior reduction in viability. To assess biocompatibility, CMSP was evaluated in normal L929 fibroblast cells at concentrations ranging from 0 to 100 μg/mL, maintaining cell viability above 80 % across all tested doses, thereby indicating favorable cytocompatibility (Fig. S5). This enhanced effect can be attributed to the combined action of SOF inhibiting cell activity and the improved internalization efficiency facilitated by the 4T1 protein coating. However, at higher concentrations (80–140 μg/mL), the difference in efficacy between CMS and CMSP became less pronounced, as cellular uptake approached saturation, thereby diminishing the additional targeting advantage of the protein coating. Live/dead staining assays further demonstrated that CM, CMS, and CMSP exhibit strong capabilities in suppressing cell proliferation, with the relatively unchanged PI fluorescence in CMSP-treated cells reflecting its anti-proliferative rather than membrane-disruptive action, consistent with a cytostatic mechanism. As clearly observed from the cell spreading area and overall morphology, although the materials do not immediately kill the cells, they effectively suppress proliferation. This leads to a morphological transformation of the cells from normally spread spindle-like shapes to spherical forms, accompanied by reduced protrusions (Fig. 2b). To further quantify the antiproliferative effect and distinguish between cytostatic and cytotoxic outcomes, a trypan blue exclusion assay was conducted. As shown in Fig. S6, CMSP treatment led to a significant decrease in the number of viable 4T1 cells compared to CM, CMS, and the control group. These results provide direct quantitative evidence of reduced cell viability, further corroborating the proliferation-inhibitory effect of CMSP observed in the CCK-8 assay and morphological studies. To directly assess the antiproliferative effects of the different formulations at the cellular level, EdU staining was performed to evaluate DNA synthesis activity in 4T1 cells. As shown in Fig. S7, strong EdU fluorescence was observed in the control group, indicating high proliferative activity. In contrast, the EdU signal gradually decreased in the CM and CMS groups, and was most markedly suppressed in the CMSP group. Flow cytometry analysis of cell viability (Fig. 2c and e) showed that CM, CMS, and CMSP treatments resulted in decreased proportions of viable cells, with death rates of 17.77 %, 26.12 %, and 40.17 %, respectively, compared to 6.39 % in the control group. It is worth noting that this apparent increase in the percentage of dead cells may partly reflect a reduction in total cell number due to proliferation inhibition, in addition to modest induction of cell death, particularly in the CMSP group.

Furthermore, colony formation assays demonstrated the long-term inhibitory effects of the materials on the proliferative capacity of 4T1 cells. Compared to the control group, the CM, CMS, and CMSP groups exhibited a progressive reduction in both the number and size of colonies, confirming effective suppression of cell proliferation and delayed tumor growth (Fig. 2d). To dynamically evaluate the inhibition of cell proliferation, a fluorescent cell proliferation dye was utilized. This dye uniformly labels intracellular proteins; during cell division, it is evenly distributed to daughter cells, resulting in a proportional decrease in fluorescence intensity with each generation. Monitoring the decline in fluorescence intensity enables the assessment of cell division frequency and proliferation rate. Flow cytometry analysis showed a more pronounced fluorescence intensity shift in the CM, CMS, and CMSP groups compared to the control (Fig. 2f and g), indicating fewer cell divisions and a reduced proliferative capacity in treated cells. These results are consistent with those obtained from the colony formation assay. Collectively, these findings demonstrate that CMSP effectively inhibits 4T1 cell viability and suppresses tumor cell proliferation.

**Fig. 3:**
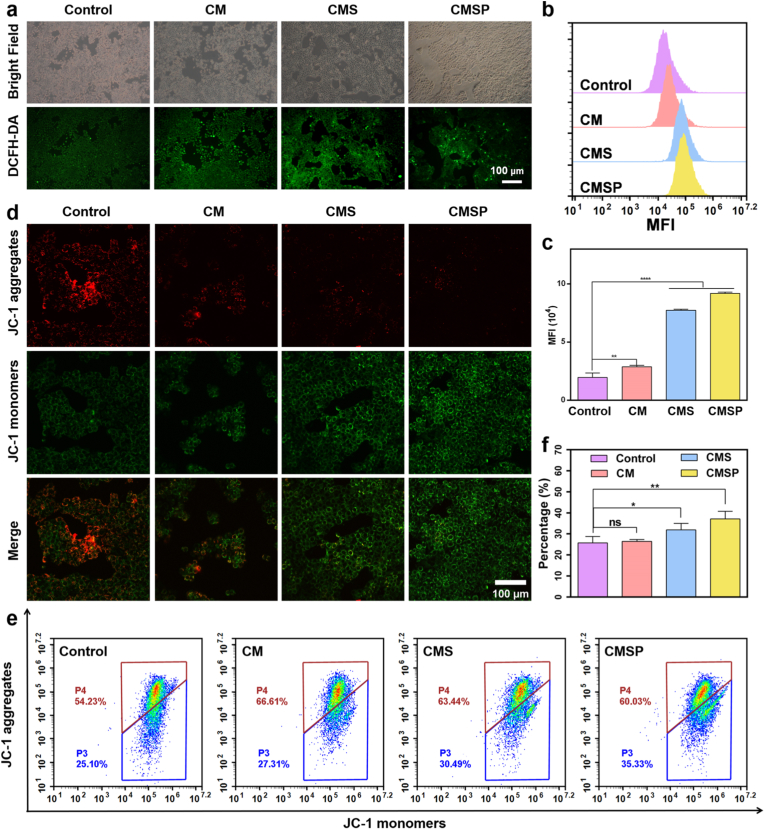
CMSP induces cellular damage by disrupting mitochondria function. (a) Fluorescence images of intracellular ROS content in 4T1 cells after incubation with CM, CMS and CMSP for 12 h. (b) Flow cytometry analysis and (c) corresponding quantitative statistics of intracellular ROS. (d) Fluorescence images of mitochondrial membrane potential after incubation of 4T1 with CM, CMS and CMSP for 12 h. JC-1 monomer (green) and JC-1 polymer (red). (e) Flow cytometry analysis and (f) corresponding quantitative statistics of mitochondrial membrane potential. Data represent mean ± SD, n = 3, ns: not significant, ∗*p* < 0.05, ∗∗*p* < 0.01, ∗∗∗∗*p* < 0.0001. (For interpretation of the references to colour in this figure legend, the reader is referred to the Web version of this article.)

Subsequently, intracellular ROS levels of 4T1 cells after treated by CM, CMS, and CMSP were assessed using an ROS-sensitive fluorescent probe. Both fluorescence imaging and flow cytometry analysis demonstrated a progressive increase in fluorescence intensity within 4T1 cells after 12 h of co-incubation with CM, CMS, and CMSP, compared to the control group (Fig. 3a–c). This indicates that CMSP effectively elevate intracellular ROS levels, thereby disrupting cellular redox homeostasis. Mitochondrial membrane potential (ΔΨm) serves as a critical parameter for evaluating mitochondrial function, as changes in ΔΨm directly reflect mitochondrial energy metabolism and the physiological state of the cell. To investigate the impact of CM, CMS, and CMSP on 4T1 cell metabolism and energy supply, ΔΨm was measured. As shown in Fig. 3d, control cells exhibited strong red fluorescence, indicating high ΔΨm and normal mitochondrial function. In contrast, 4T1 cells treated with CM, CMS, and CMSP displayed a gradual decrease in red fluorescence intensity along with a corresponding increase in green fluorescence, suggesting a reduction in ΔΨm. This implies that material treatment significantly impairs mitochondrial function. Consistent with these observations, flow cytometry analysis (Fig. 3e) and corresponding quantitative statistics (Fig. 3f) revealed a progressive increase in the proportion of cells exhibiting green fluorescence (from 25.10 % to 35.33 %), corresponding to the monomeric form of the probe, further supporting the loss of mitochondrial membrane potential. Collectively, these results demonstrate that the developed CMSP system effectively targets mitochondria, disrupts their normal function, and thereby induces cellular damage.

**Fig. 4:**
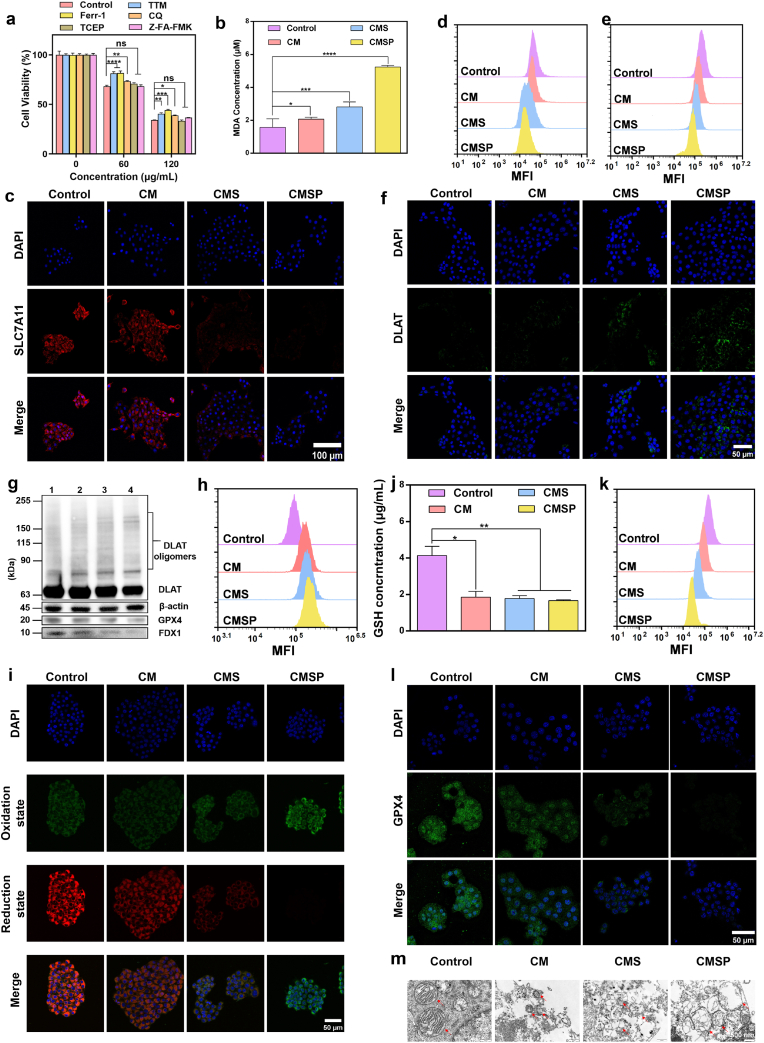
CMSP induce ferroptosis and cuproptosis. (a) Cell survival rate of 4T1 cells after being incubated with TTM, Ferr-1, CQ, TCEP and Z-FA-FMK for 6 h and CMSP for 24 h, respectively. (b) The GSH content in 4T1 cells after incubation with CM, CMS and CMSP for 12 h. (c) Fluorescence imaging and (d) flow cytometry analysis of the SLC7A11 protein expression in 4T1 cells after incubation with CM, CMS and CMSP for 12 h. (e) Flow cytometry analysis of FDX1 protein expression in 4T1 cells incubated with CM, CMS and CMSP for 12 h. (f) Fluorescence images of DLAT protein aggregation in 4T1 cells incubated with CM, CMS and CMSP for 12 h. (g) Western blot images of DLAT, GPX4 and FDX1. (h) Flow cytometry analysis and (i) fluorescence imaging of lipid ROS in 4T1 cells after incubation with CM, CMS and CMSP for 12 h. (j) The intracellular MDA content in 4T1 cells after incubation with CM, CMS and CMSP for 12 h. (k) Flow cytometry analysis and (l) fluorescence imaging of GPX4 protein expression in 4T1 cells incubated with CM, CMS and CMSP for 12 h. (m) TEM images of mitochondrial ultrastructure in 4T1 cells after different treatments. The red arrow indicates the mitochondria. Data represent mean ± SD, n = 3, ns: not significant, ∗*p* < 0.05, ∗∗*p* < 0.01, ∗∗∗*p* < 0.001, ∗∗∗∗*p* < 0.0001. (For interpretation of the references to colour in this figure legend, the reader is referred to the Web version of this article.)

Next, we investigated the mechanism by which the CMSP nanoassembler induce tumor cell death. Cells were treated with specific inhibitors targeting distinct cell death pathways: CQ (autophagy inhibitor), TTM (cuproptosis inhibitor), Ferr-1 (ferroptosis inhibitor), Z-FA-FMK (apoptosis inhibitor), and TCEP (disulfidptosis inhibitor). Groups treated solely with each inhibitor served as controls to account for any potential cytotoxic effects of the inhibitors themselves. The differences in cell viability between the treatment groups and their respective inhibitor-only controls were then compared. The results showed that only co-treatment with TTM or Ferr-1 significantly reversed cell death, demonstrating that CMSP nanoassembler promotes tumor cell death through both cuproptosis and ferroptosis pathways (Fig. 4a).

CMSP incorporates SOF, a multi-kinase inhibitor known to suppress System Xc^−^ activity, thereby reducing cystine uptake and subsequently impairing GSH synthesis. Additionally, copper ions released from the degraded Cu-MOF further consume intracellular GSH. Therefore, we measured both oxidized glutathione (GSSG) and total glutathione levels. The results confirmed a significant decrease in intracellular GSH content following treatment with CMSP (Fig. 4b). Furthermore, immunofluorescence staining demonstrated that SOF effectively suppressed SLC7A11 expression (Fig. 4c–d, Fig. S8). Compared to the control group, the CM, CMS, and CMSP groups also exhibited significantly downregulated FDX1 expression (Fig. 4e, Fig. S9), a marked decrease in DLAT monomer levels, and a progressive increase in DLAT oligomers (Fig. 4f). These findings indicate that the CMSP nanoassembler effectively inhibits FDX1 expression and promotes DLAT oligomerization. Western blot analysis corroborated these findings (Fig. 4g), collectively confirming the ability of CMSP to induce cuproptosis.

Detection of lipid peroxidation serves as a key indicator for confirming ferroptosis. The extent of lipid peroxidation in 4T1 cells treated with CM, CMS, or CMSP was assessed using the lipid peroxidation probe BODIPY C11. Flow cytometry quantification of the oxidized state (BODIPY 581/591 C11) revealed a progressive increase in fluorescence intensity in the treated groups (Fig. 4h, Fig. S10). Fluorescence imaging showed the highest green fluorescence intensity (indicative of oxidation) and the lowest red fluorescence intensity in the CMSP group (Fig. 4i), further supporting the induction of lipid peroxidation.

Polyunsaturated fatty acids (PUFAs) are oxidized under the catalytic action of iron and ROS, generating lipid peroxidation products such as malondialdehyde (MDA) and 4-hydroxynonenal (4-HNE). Quantification of intracellular MDA levels provides a reliable measure of lipid peroxidation severity and confirms the occurrence of ferroptosis. As shown in Fig. 4j, MDA levels progressively increased in cells treated with CM, CMS, and CMSP, providing further evidence of lipid peroxidation. GPX4, a GSH-dependent antioxidant enzyme that reduces lipid hydroperoxides to non-toxic lipid alcohols and thereby inhibits the accumulation of lipid peroxidation, is a key marker for ferroptosis. GPX4 expression was evaluated *via* western blotting and flow cytometry (Fig. 4k–l, Fig. S11). The results showed significant downregulation of GPX4 expression in the CM, CMS, and CMSP groups compared to the control, a trend clearly evident in the western blot data. This confirms that the CMSP nanoassembler effectively induces ferroptosis. Moreover, TEM revealed progressive mitochondrial damage across treatment groups (Fig. 4m). While control cells displayed intact mitochondrial membranes and cristae, CM and CMS treatments led to partial cristae loss and membrane disruption. CMSP-treated cells exhibited the most severe mitochondrial swelling and cristae collapse, indicative of profound mitochondrial dysfunction. Taken together, these results suggest that CMSP promotes both ferroptosis and cuproptosis through convergent redox disruption and mitochondrial dysregulation. The observed depletion of GSH and NADPH, along with lipid peroxidation and DLAT oligomerization, points toward a unified stress response that engages both pathways. Although mechanistically distinct, ferroptosis and cuproptosis may be co-activated in a mutually reinforcing manner under CMSP treatment, potentially amplifying the overall anti-proliferative effect.

### 3.3 *In vivo* antitumor efficiency

**Fig. 5:**
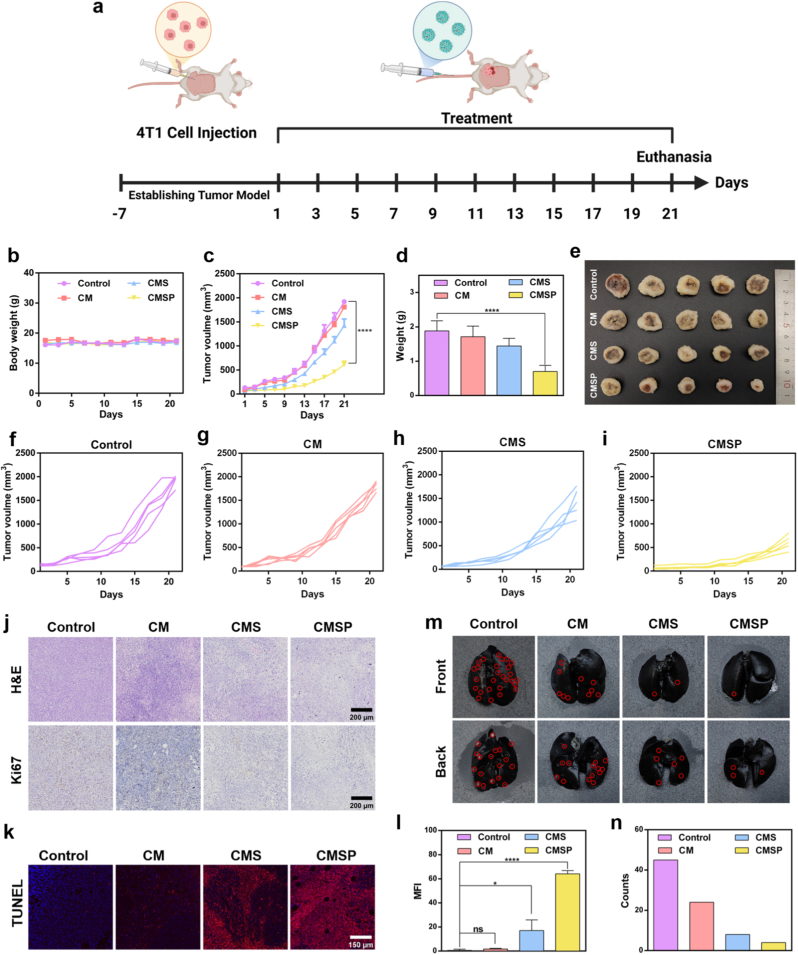
Antitumor efficiency in mice bearing 4T1 tumors treated with CM, CMS and CMSP. (a) Schematics depicting the therapeutic schedule in subcutaneous 4T1 cancer with intravenous injection of saline, CM, CMS or CMSP. Schemes created with BioRender.com. (b) Changes in the body weight of mice. (c) Changes in tumor volume. (d) Weight statistics of tumor tissues. (e) Images of tumor. (f–i) Tumor volume changes of saline, CM, CMS and CMSP treatment. (j) H&E and Ki67 staining of tumor tissue in saline, CM, CMS, and CMSP treatment groups. (k) TUNEL staining and (l) fluorescence statistics of tumor tissue in saline, CM, CMS, and CMSP treatment groups. (m) Images of India Ink staining in the lungs of mice implanted with tumor models and (n) results of lung metastases. Data represent mean ± SD, n = 3, ns: not significant, ∗*p* < 0.05, ∗∗∗∗*p* < 0.0001.

Next, we established a 4T1 cell-derived breast cancer mouse tumor model to evaluate the *in vivo* antitumor efficacy of the CMSP nanoassembler (Fig. 5a). To evaluate the *in vivo* biodistribution behavior of CMSP, 4T1 tumor-bearing mice were injected with ICG-labeled CMS or CMSP nanoparticles, followed by fluorescence imaging at 12 h post-injection. As shown in Fig. S12a, both formulations accumulated at the tumor site to a similar extent. However, CMS-treated mice exhibited broader systemic fluorescence, indicating a more diffuse biodistribution and greater off-target accumulation. In contrast, CMSP showed more confined fluorescence localization, with reduced background signal in non-tumor regions. *Ex vivo* imaging of excised organs and tumors (Fig. S12b–c) further confirmed that CMSP achieved enhanced tumor selectivity, likely due to improved immune evasion and homotypic targeting conferred by the tumor lysate coating. Saline, CM, CMS, and CMSP were administered to tumor-bearing mice *via* tail vein injection, and changes in body weight and tumor volume were systematically monitored. During the treatment period, no significant alterations in body weight were observed among the groups (Fig. 5b), indicating minimal systemic toxicity. However, notable differences in tumor volume were evident across the treatment groups (Fig. 5c and 5f–i). The saline group exhibited rapid tumor growth over the 21-day observation period, demonstrating negligible antitumor activity. The CM group also showed limited therapeutic efficacy. In contrast, CMS, which was loaded with SOF, exhibited significantly enhanced tumor suppression compared to CM. Notably, CMSP, which was coated with whole tumor cell membrane proteins, displayed the most potent antitumor effect among all groups. This enhanced therapeutic performance is likely due to the presence of tumor-specific antigens or receptors on the nanoparticle surface, enabling homotypic tumor cell recognition and binding for precise targeting. As a result, CMSP achieved greater accumulation at the tumor site, leading to higher local drug concentrations and superior therapeutic outcomes. Tumor growth was visually documented throughout the treatment period (Fig. S13). At the end of the experiment, tumors were excised, photographed, and weighed (Fig. 5d and e), with the results closely mirroring those of the tumor growth curves. Collectively, these findings confirm that CMSP nanoassembler effectively inhibits tumor growth.

Histopathological analysis of tumor tissues was performed using H&E and Ki67 staining (Fig. 5j). The saline-treated group exhibited dense cellular architecture and active tumor growth, whereas the treatment groups showed reduced cancer cell density, less nuclear integrity, and a more disorganized tissue structure. Ki67, a nuclear protein closely associated with cell proliferation, serves as a reliable marker for assessing tumor cell proliferation. A higher number of Ki67-positive cells indicate greater proliferative activity. Immunostaining revealed the highest density of brown-stained (Ki67-positive) cells in the saline group and the lowest in the CMSP group, consistent with the H&E findings. TUNEL staining, which specifically labels fragmented DNA in apoptotic cells, was used to assess apoptosis. As shown in Fig. 5k and l, the CMSP group exhibited significantly higher red fluorescence intensity compared to other groups, indicating the highest rate of apoptosis. These results demonstrate that CMSP nanoassembler effectively suppresses tumor cell proliferation and promotes apoptosis.

Tumor metastasis is a critical indicator of cancer progression. Given that the lungs are common sites of metastasis, evaluating lung metastases provides an accurate reflection of tumor dissemination. India ink staining was employed to visualize metastatic nodules in lung tissues (Fig. 5m). The saline-treated group exhibited numerous white metastatic nodules on both lung surfaces, whereas the CMSP group showed the fewest metastatic lesions. Quantitative analysis (Fig. 5n) confirmed statistically significant differences between groups, further supporting the antimetastatic efficacy of CMSP. These findings indicate that CMSP nanoassembler effectively inhibits tumor metastasis.

**Fig. 6:**
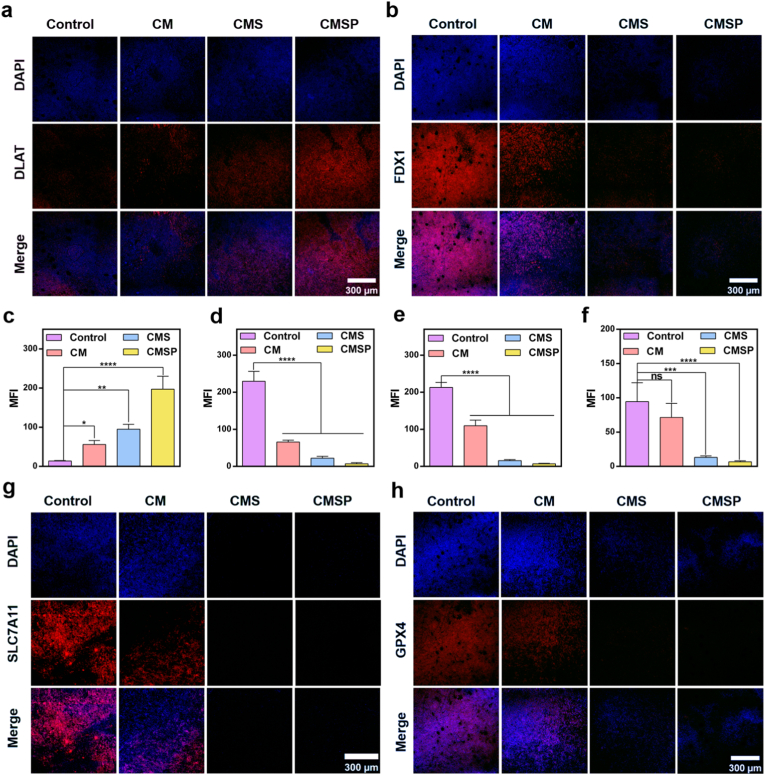
CMSP induce ferroptosis and cuproptosis in mice bearing 4T1 tumors. Fluorescence staining of (a) DLAT, (b) FDX1, and fluorescence statistics of (c) DLAT, (d) FDX1; Fluorescence staining of (g) SLC7A11 and (h) GPX4 and fluorescence statistics of (e) SLC7A11 and (f) GPX4. (n = 3, ns: not significant, ∗*p* < 0.05, ∗∗*p* < 0.01, ∗∗∗*p* < 0.001, ∗∗∗∗*p* < 0.0001).

Next, we validated the antitumor mechanism of the CMSP nanoassembler *in vivo*. Protein immunofluorescence staining was conducted on mouse tumor tissues to assess expression changes in key proteins associated with cuproptosis and ferroptosis. As shown in Fig. 6a–d, FDX1, a critical regulator of cuproptosis, was significantly downregulated, and DLAT, a core enzyme of the pyruvate dehydrogenase complex, exhibited prominent oligomerization. Simultaneously, Fig. 6e–h revealed a marked reduction in the expression levels of SLC7A11 and GPX4, which are established biomarkers of ferroptosis. Collectively, these results demonstrate that the drug delivery system effectively induces both cuproptosis and ferroptosis, thereby promoting tumor cell death.

Finally, the hemocompatibility of CM, CMS, and CMSP was evaluated using a hemolysis assay. After incubating CM, CMS, and CMSP with blood at specified ratios, all materials exhibited hemolysis rates below 5 % across all tested concentrations (Fig. S14). This demonstrates excellent hemocompatibility and a minimal risk of inducing hemolysis. Subsequently, CM, CMS, and CMSP solutions were administered *via* tail vein injection. Blood samples were collected at defined time points to assess liver function, kidney function, and hematological parameters. The results showed that all measured indices in both the control and treatment groups remained within normal physiological ranges (Figs. S15–16), further confirming the favorable biocompatibility and safety profile of CMSP. Additionally, histopathological examination of major organs (heart, liver, spleen, kidney, lung) from mice after 21 days of treatment revealed well-preserved tissue morphology and cellular architecture in the CM-, CMS-, and CMSP-treated groups, with no significant differences compared to the control group (Fig. S17). Notably, a marked reduction in pulmonary nodules was observed in the lung tissues of the treated groups. Collectively, these findings indicate that CM, CMS, and CMSP do not induce detectable organ toxicity.

## 4 Conclusion

This study successfully developed a SOF-loaded Cu-MOF nanoplatform coated with whole tumor cell lysate. The nanoplatform selectively degrades in the weakly acidic TME, releasing Cu^2+^ ions that are subsequently reduced to Cu^+^ by intracellular GSH. The generated Cu^+^ ions activate dual cell death pathways: (i) ferroptosis, induced *via* a Fenton-like reaction that catalyzes ROS production and leads to the accumulation of lethal lipid peroxidation products; and (ii) cuproptosis, triggered by Cu^+^ binding to lipoylated proteins in the TCA cycle (e.g., DLAT), resulting in abnormal oligomerization and inhibition of iron-sulfur cluster protein synthesis. Concurrently, the co-released SOF enhances both pathways by inhibiting System Xc^−^, thereby reducing GSH synthesis and weakening antioxidant defenses, as well as decreasing the chelation of Cu^+^ by GSH. These effects synergistically amplify the cuproptosis-ferroptosis cascade in a bidirectional manner. Both *in vitro* and *in vivo* experiments using the 4T1 breast cancer model demonstrated that CMSP nanoassembler significantly suppresses tumor proliferation, promotes apoptosis, and inhibits metastasis, while maintaining excellent biosafety. Collectively, this multi-mechanistic cooperative strategy offers a promising approach for metal ion-mediated regulation of cell death in cancer therapy.

## CRediT authorship contribution statement

**Mingyue Zhang:** Methodology, Investigation, Formal analysis, Data curation. **Ke Zhang:** Methodology, Formal analysis. **Shiyao Guo:** Visualization, Investigation, Formal analysis. **Peiran Chen:** Visualization, Validation, Methodology. **Bangliu Yang:** Visualization, Validation. **Xueqian Wang:** Methodology, Investigation. **Xiaotong Lu:** Methodology, Investigation, Formal analysis. **Yuhong Zhuo:** Validation, Investigation. **Shaofeng Chen:** Validation, Methodology, Formal analysis. **Dongqin Yu:** Writing – original draft, Funding acquisition, Formal analysis. **Lian-Hua Fu:** Resources, Funding acquisition. **Chao Qi:** Writing – review & editing, Supervision, Project administration, Funding acquisition, Conceptualization. **Kaiyong Cai:** Supervision, Project administration, Funding acquisition.

## Declaration of competing interest

The authors declare that they have no known competing financial interests or personal relationships that could have appeared to influence the work reported in this paper.

## Data Availability

Data will be made available on request.
