# Supplementary material for: Tumor lysate-cloaked CuMOF-sorafenib nanoassembler: A synergistic cuproptosis-ferroptosis nanoweapon against tumors
Source: Mater Today Bio. 2025 Dec 5;36:102623. doi: 10.1016/j.mtbio.2025.102623 (PMC12753271; doi:10.1016/j.mtbio.2025.102623)
Supplement: Multimedia component 1 [file mmc1.docx]

Supporting Information

Tumor Lysate-Cloaked CuMOF-Sorafenib Nanoassembler: A Synergistic Cuproptosis-Ferroptosis Nanoweapon Against Tumors

Mingyue Zhang^a,^ ^#^, Ke Zhang^a, #^, Shiyao Guo^a^, Peiran Chen^a^, Bangliu Yang^a^, Xueqian Wang^a^, Xiaotong Lu^a^, Yuhong Zhuo^a^, Shaofeng Chen^a^, Dongqin Yu^a,^ *, Lian-Hua Fu^b,^ *, Chao Qi^a,^ *, Kaiyong Cai^a,^ *

^a^ Key Laboratory of Biorheological Science and Technology, Ministry of Education, College of Bioengineering, Chongqing University, Chongqing, 400044, China

^b^ School of Biomedical Engineering, Shenzhen University Medical School, Shenzhen University, Shenzhen 518055, China

^#^ These authors contributed equally to this work.

**^*^Corresponding author:** yu_dongqin89@163.com; fulianhua1990@163.com; qichao2020@cqu.edu.cn; kaiyong_cai@cqu.edu.cn

**Supporting Figures**


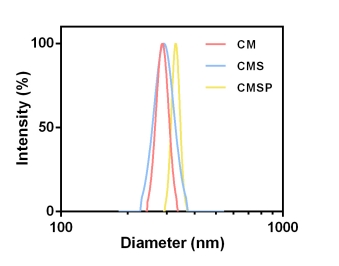


**Fig. S1**. Hydrodynamic size distribution of CM, CMS and CMSP.


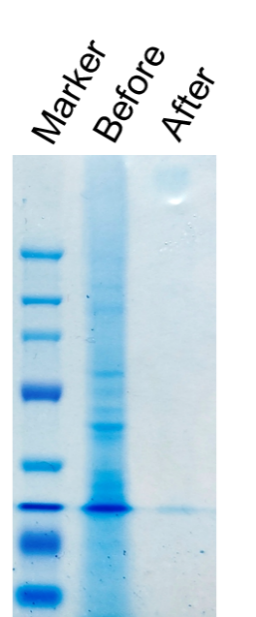


**Fig. S2.** SDS-PAGE analysis of 4T1 tumor lysate before and after loading into CMSP nanoparticles.


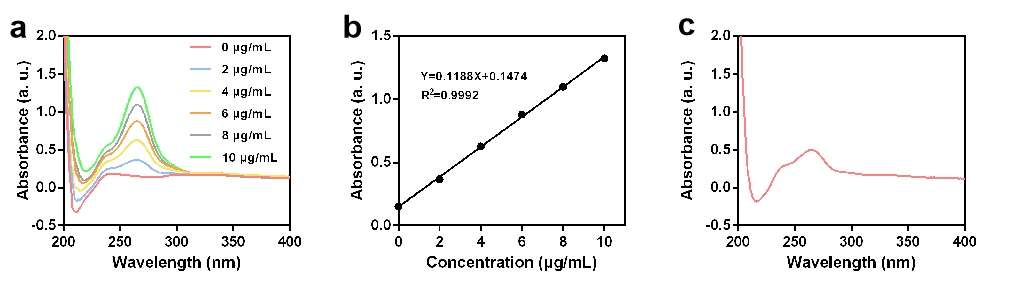


Fig. S3. (a) UV-vis absorption of methanol solutions with different concentrations of sorafenib. (b) Sorafenib standard curve. (c) The UV absorption spectrum was obtained from the supernatant, which was diluted 200 times following centrifugal separation of the drug-loaded sample.


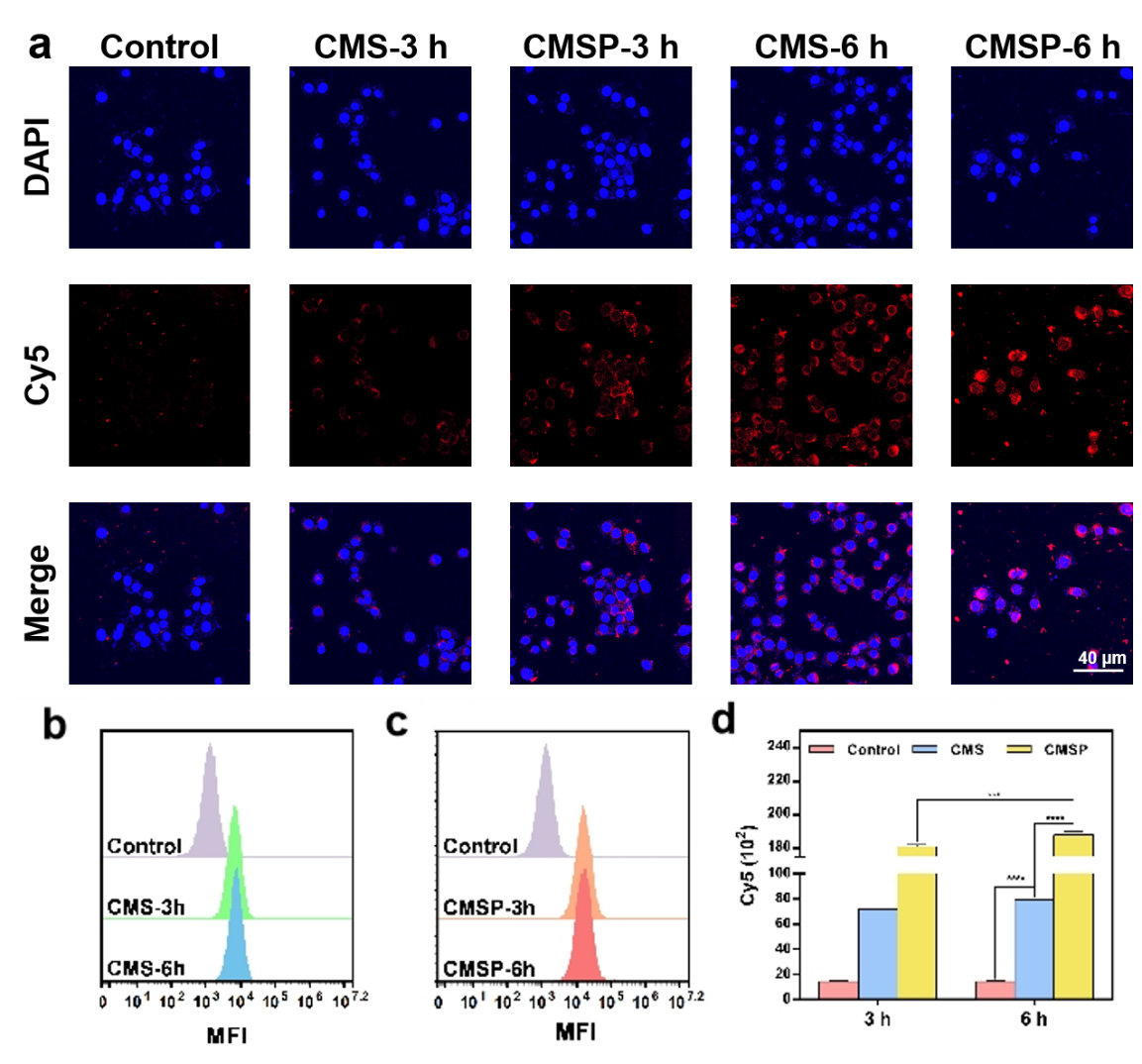


**Fig. S4**. (a) CLSM images of nuclei (DAPI, blue) and Cy5-labed materials (red) after 4T1 cells were co-incubated with different materials for 3 and 6 h. Flow cytometry analysis of 4T1 cells co-incubated with (b) CMS and (c) CMSP for 3 h and 6 h. (d) Quantitative statistical analysis of flow cytometry results from panels (b) and (c). Data represent mean ± SD, n = 3, ****p* < 0.001, *****p* < 0.0001.


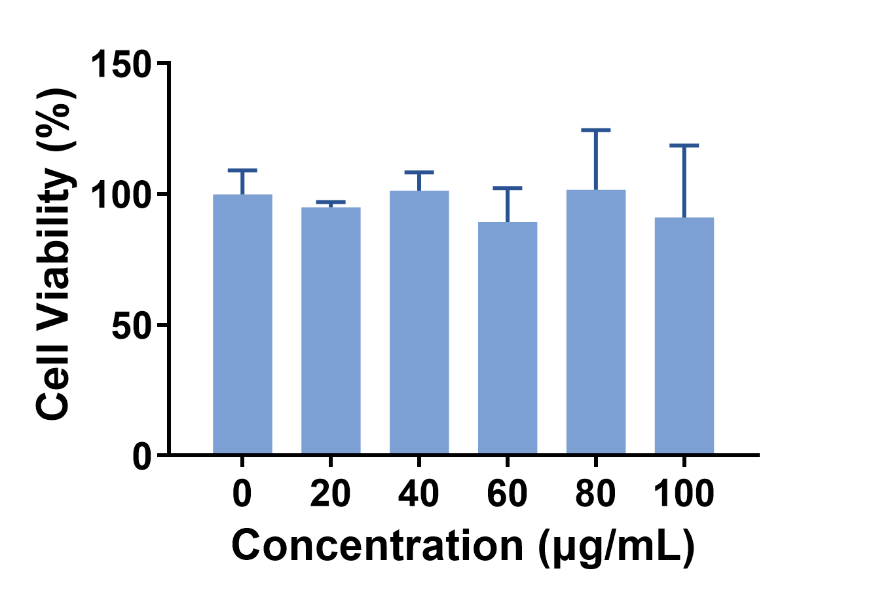


**Fig. S5.** Cell viability of L929 cells after treatment with CMSP at different concentrations.

**Fig. S6.** Quantification of viable 4T1 cells after treatment with CM, CMS, and CMSP using trypan blue exclusion assay. Data are expressed as mean ± SD (n = 3). *****p*< 0.0001.


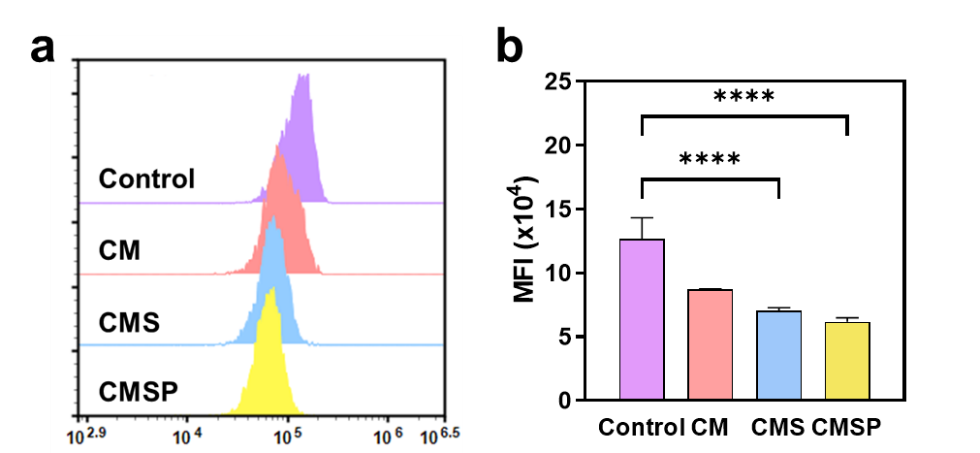


**Fig. S7.** EdU staining analysis of cell proliferation after treatment with CM, CMS, and CMSP (100 μg/mL) for 24 h. (a) Representative fluorescence histograms of EdU incorporation. (b) Quantitative analysis of EdU fluorescence. Data are expressed as mean ± SD (n = 3). *****p* < 0.0001.


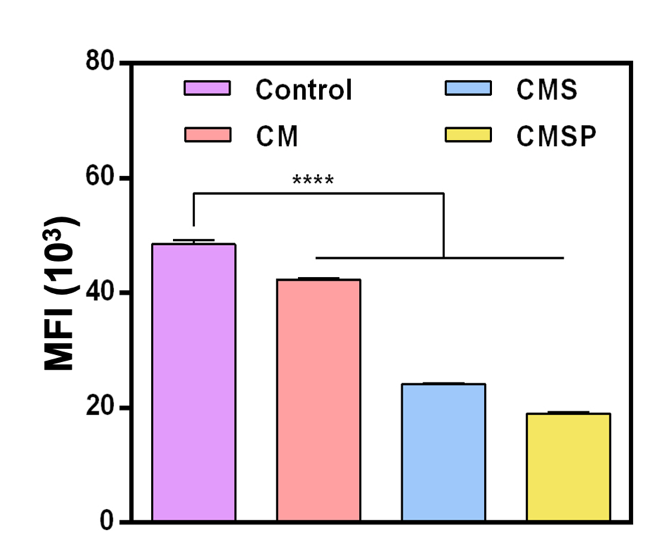


**Fig. S8**. Flow cytometry quantitative statistics of the SLC7A11 protein expression in 4T1 cells. Data represent mean ± SD, n=3, *****p* < 0.0001.


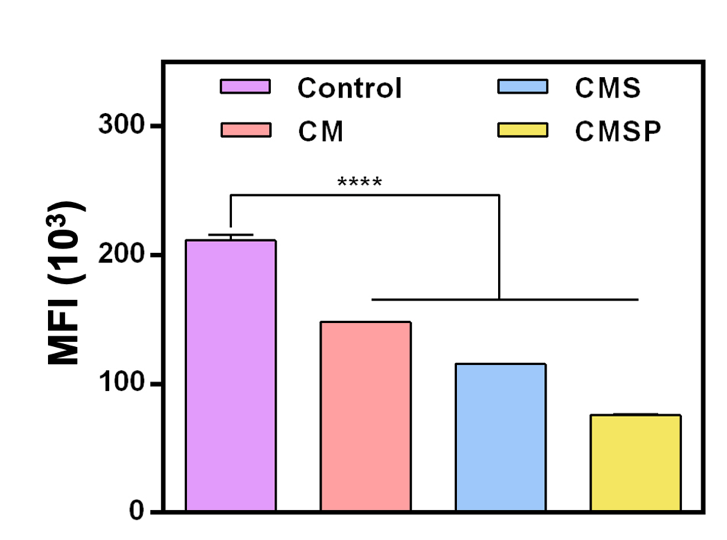


**Fig. S9**. Flow cytometry quantitative statistics of FDX1 protein expression in 4T1 cells. Data represent mean ± SD, n=3, *****p* < 0.0001.


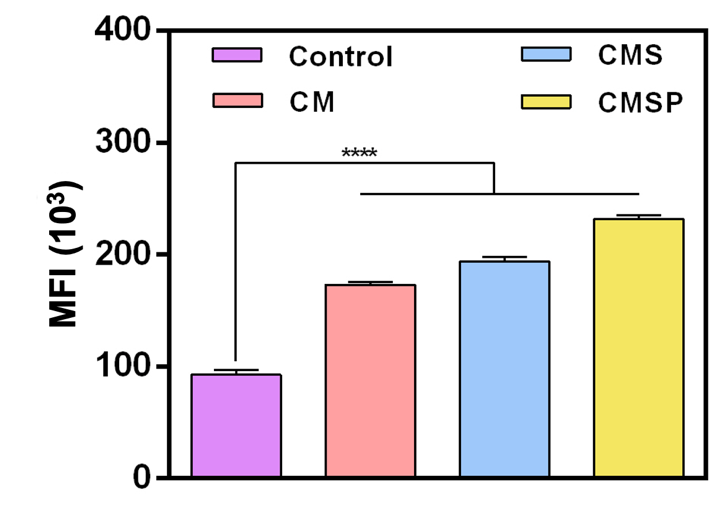


**Fig. S10**. Flow cytometry quantitative statistics of lipid ROS in 4T1 cells. Data represent mean ± SD, n=3, *****p* < 0.0001.


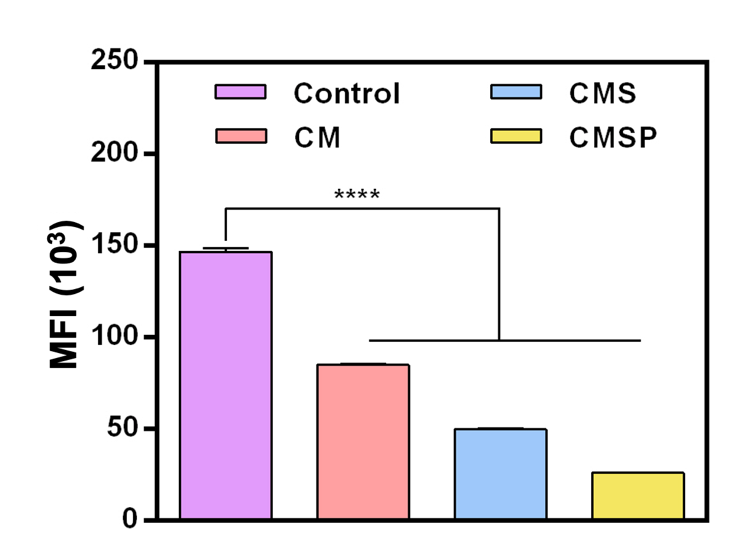


**Fig. S11**. flow cytometry quantitative statistics of GPX4 protein expression in 4T1 cells. Data represent mean ± SD, n=3, *****p* < 0.0001.


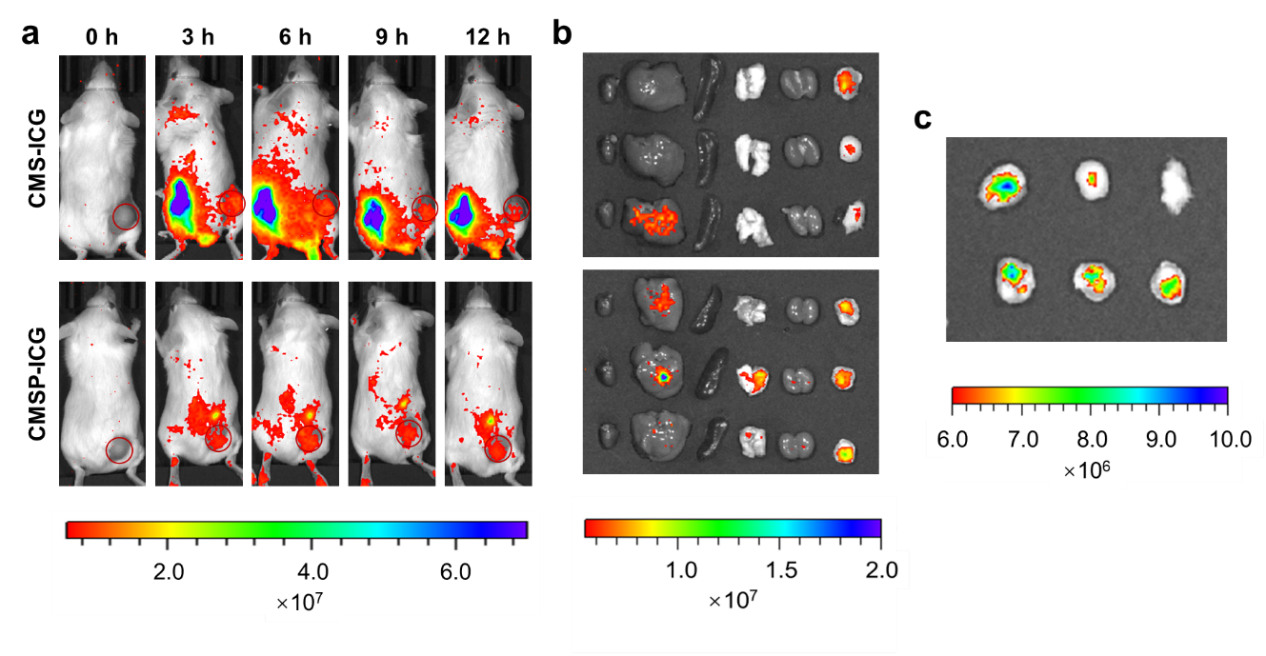


**Fig. S12.** *In vivo* biodistribution of CMS and CMSP nanoparticles labeled with ICG. (a) Fluorescence images of whole-body distribution at 0, 3, 6, 9, 12 h post-injection. (b) *Ex vivo* fluorescence images of major organs and tumors collected from mice. (c) *Ex vivo* fluorescence images of tumors collected from mice (n = 3).


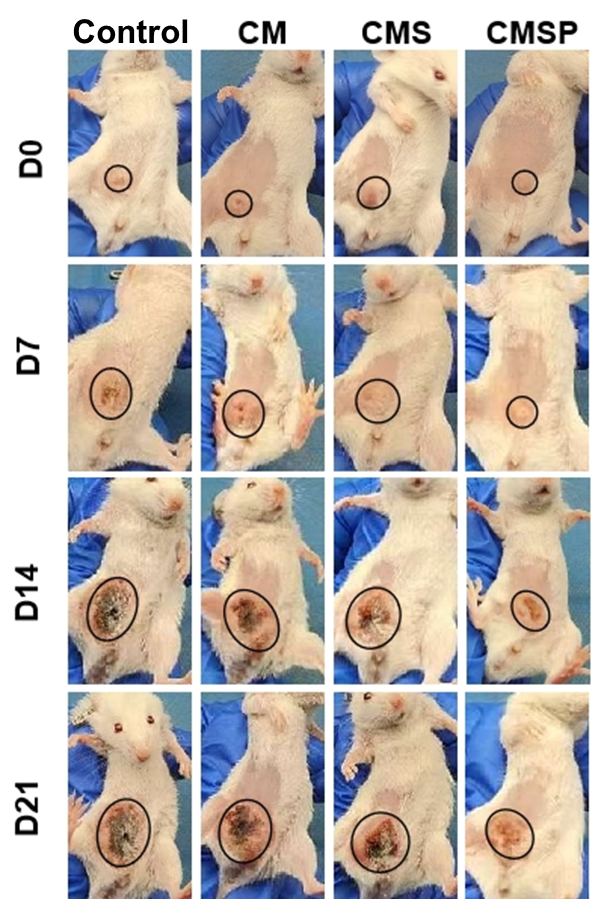


**Fig. S13**. Representative photographic images showing tumor volume progression in mice on days 0, 7, 14, and 21.


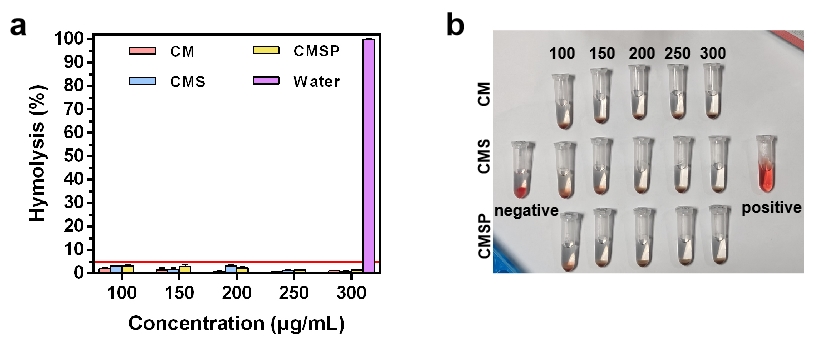


**Fig. S14**. *In vitro* hemolysis (a) and images (b) of CM, CMS and CMSP.


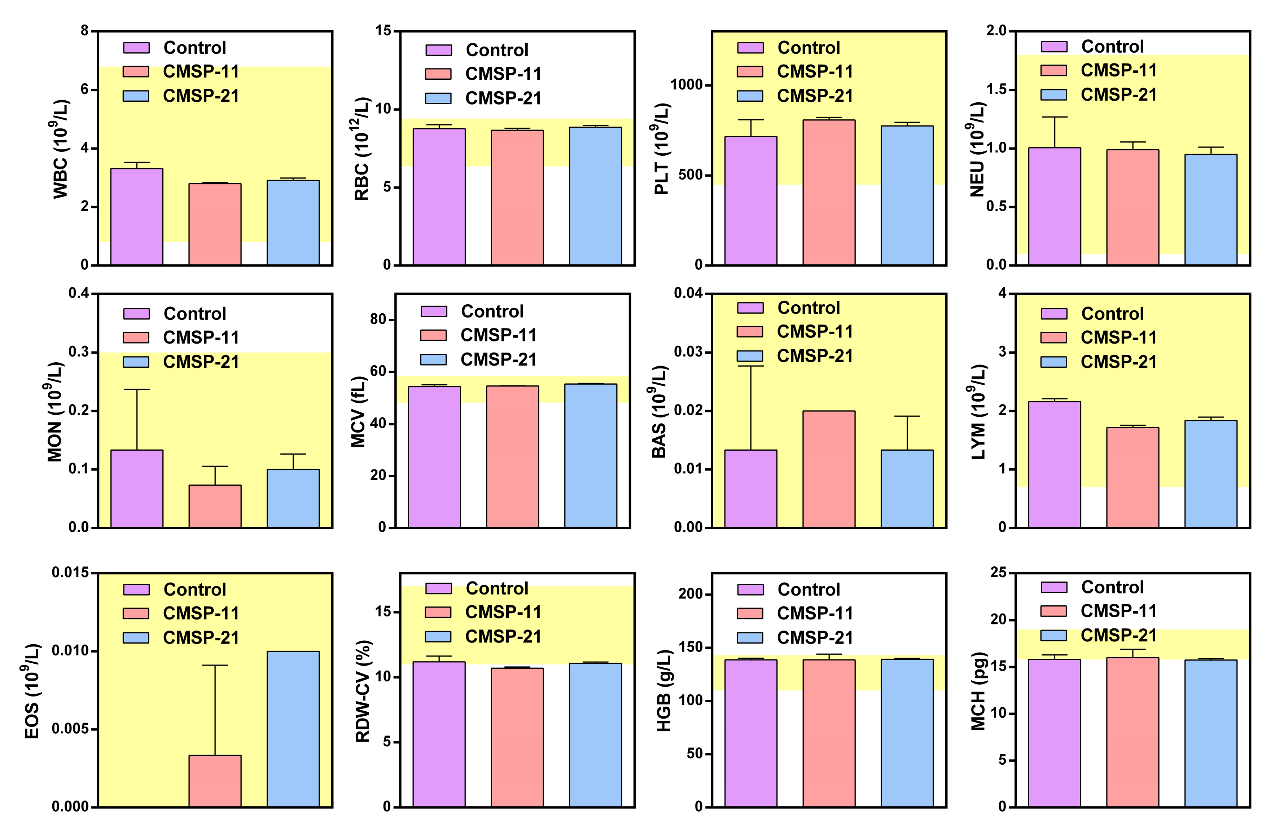


**Fig. S15**. Determination of the main indexes of mouse blood routin, yellow shading: reference standard for biochemical indicators.


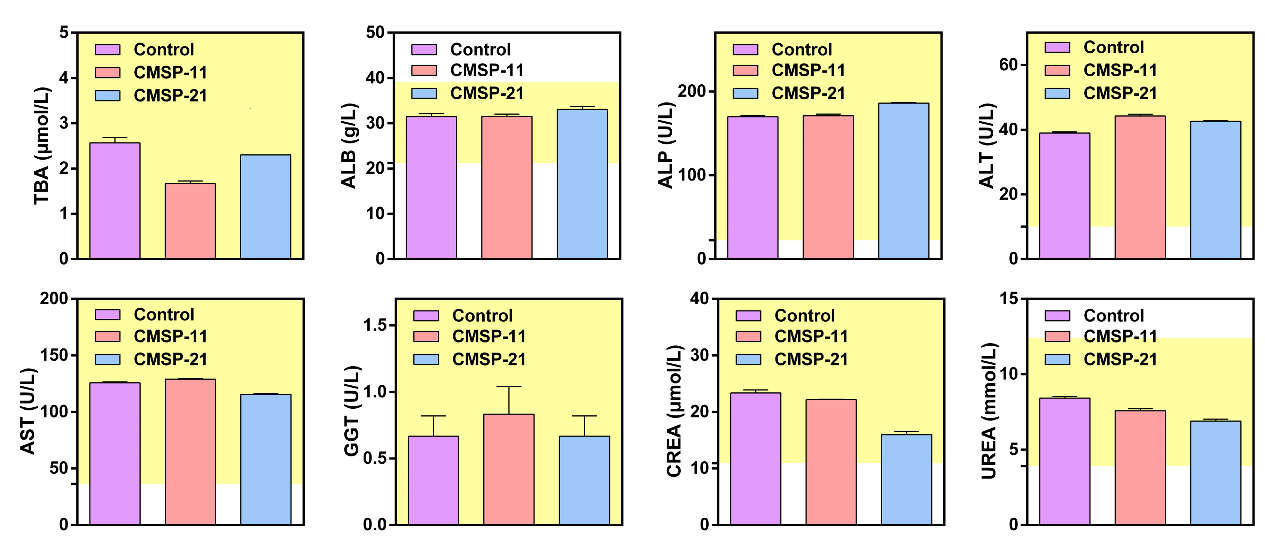


**Fig. S16**. Determination of main indicators of mouse liver and kidney function, yellow shading: reference standard for biochemical indicators.


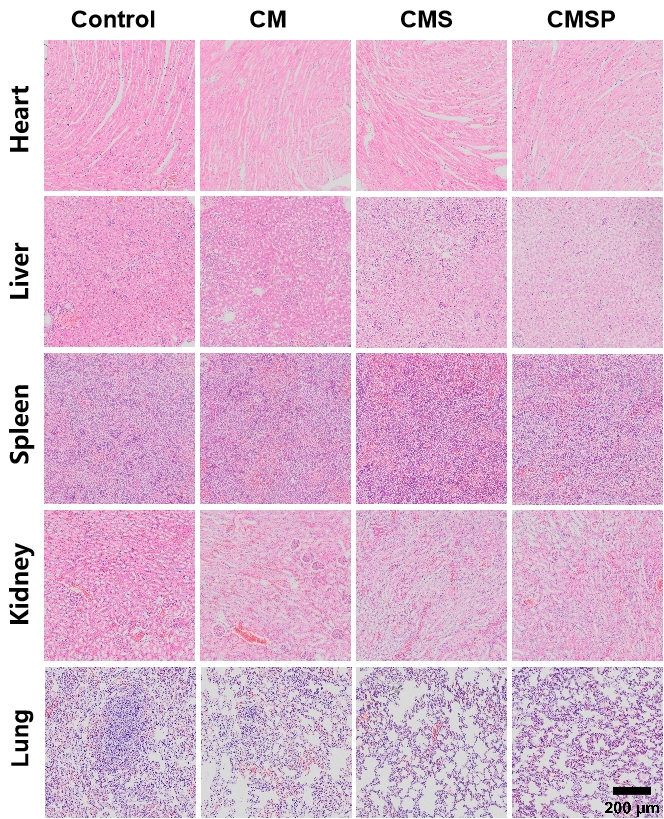


**Fig. S17**. H&E staining of the heart, liver, spleen, kidney, and lungs of mice treated with Saline, CM, CMS and CMSP.
